# A simple approach for protein name identification: prospects and limits

**DOI:** 10.1186/1471-2105-6-S1-S15

**Published:** 2005-05-24

**Authors:** Katrin Fundel, Daniel Güttler, Ralf Zimmer, Joannis Apostolakis

**Affiliations:** 1 Institut für Informatik, Ludwig-Maximilians-Universität München, Amalienstrasse 17, 80333 München, Germany

## Abstract

**Background:**

Significant parts of biological knowledge are available only as unstructured text in articles of biomedical journals. By automatically identifying gene and gene product (protein) names and mapping these to unique database identifiers, it becomes possible to extract and integrate information from articles and various data sources.

We present a simple and efficient approach that identifies gene and protein names in texts and returns database identifiers for matches. It has been evaluated in the recent BioCreAtIvE entity extraction and mention normalization task by an independent jury.

**Methods:**

Our approach is based on the use of synonym lists that map the unique database identifiers for each gene/protein to the different synonym names. For yeast and mouse, synonym lists were used as provided by the organizers who generated them from public model organism databases. The synonym list for fly was generated directly from the corresponding organism database. The lists were then extensively curated in largely automated procedure and matched against MEDLINE abstracts by exact text matching. Rule-based and support vector machine-based post filters were designed and applied to improve precision.

**Results:**

Our procedure showed high recall and precision with F-measures of 0.897 for yeast and 0.764/0.773 for mouse in the BioCreAtIvE assessment (Task 1B) and 0.768 for fly in a post-evaluation.

**Conclusion:**

The results were close to the best over all submissions. Depending on the synonym properties it can be crucial to consider context and to filter out erroneous matches. This is especially important for fly, which has a very challenging nomenclature for the protein name identification task. Here, the support vector machine-based post filter proved to be very effective.

## Background

Protein name identification in texts is an important challenge in bioinformatics. Several approaches have been proposed to tackle this problem. Machine learning and statistical techniques proved to be useful [1-4]. Other methods focus on linguistic techniques [5], or are based on the usage of dictionaries extracted from databases, ontologies, and other data sources [6-8]. Some methods rely on the combination of dictionaries and linguistic/machine learning techniques [9]. An overview of biological named entity extraction and an introduction into the problem with description of fly synonyms is given in [10].

Yeast, mouse, and fly are interesting organisms from several points of view: they are among the experimentally most intensively studied organisms. As model organisms they are frequently used to elucidate pathways and molecular interactions that might play a role in human diseases. For each of these organisms, there exists a well curated and public organism specific database. As many scientific publications deal with these organisms, a reliable gene/protein name detection method would be a significant advance for information retrieval and extraction. Due to the differences in their protein nomenclature, the set of these three organisms is well suited for comparing gene/protein name identification tools.

When an entity extraction system is required to provide database identifiers for identified proteins, it is certainly beneficial to use a dictionary-based approach. We present a simple and efficient approach for protein name identification. It is based on extensively curated synonym lists. Synonyms are then searched within MEDLINE abstracts by exact matching. The matching tool only recognizes spellings of a synonym that are explicitly contained in the synonym list and it does not consider context for matching. Its initial main application was to test different synonym lists and to evaluate different kinds of expansions of synonym lists performed during curation. This tool allows us to rapidly evaluate modifications of synonyms and enables us to build high-quality synonym lists. These can then also be used as a prerequisite for text-mining with other text-mining tools. Additionally, we present post filters which improve precision of our results; this is important for synonyms that overlap with common words or expressions having different meanings.

The BioCreAtIvE evaluation (task 1B) [11] was set up to assess the ability of automated systems to identify names of genes and gene products and normalize them by association of a unique identifier for each gene/gene product. In this paper we use the terms gene, gene product and protein as synonyms even though these terms refer to distinct biological objects.

Our goal in participating in the BioCreAtIvE evaluation was to assess the recall and precision that can be achieved with extensively curated synonym lists and exact string-matching, and to assess the difference with more sophisticated text-mining approaches. This evaluation allows us to evaluate our approach, especially the quality of annotation, on a blind prediction basis and for an independent test set. By using comparable synonym lists, it also allows us to compare our approach to a more involved approximate matching procedure implemented in the tool ProMiner [6,12] in terms of recall and precision, as well as runtime and ease of use.

## Methods

### Generation and curation of synonym lists

The performance of an approach based on exact matching depends heavily on the quality and completeness of the synonym list used for searching. The synonym lists for yeast and mouse were created on the basis of the lists provided by the BioCreAtIvE organizers. These lists were originally extracted from the corresponding organism specific databases, Saccharomyces Genome Database (SGD) [13] and Mouse Genome Database (MGD) [14]. The fly synonym list was extracted directly from FlyBase [15] and provided by Hanisch et al. [12].

We curated the provided lists to cover additional, frequently used synonyms and remove unspecific and inappropriate synonyms. The individual curation steps are fully automated, they can be applied individually and thus the curation procedure can be adapted to the synonym list that needs to be curated. Essentially we always follow the same curation procedure, usually only very few parameters or rules are changed when it is applied to a synonym list of a different organism. Here we describe the principles and the sequential steps as applied for the mouse synonym list; for yeast and fly the procedure was slightly modified.

In a first step, synonyms consisting solely of digits and/or special characters and synonyms of length less than two are removed. Subtype specifiers are expanded to equivalent other specifiers (a⇔alpha). Special characters at the beginning or end of a synonym are removed and different spelling variants like the insertion of a hyphen or space between alphabetic characters and digits are added (Igf 1⇔Igf-1⇔Igf1). Synonyms of a length less than six characters are added in upper case and with the first character in upper case.

Eventually, organism specific expansion is performed, e.g. yeast synonyms as defined in the synonym list are often mentioned in texts with extension 'p', e.g. SOH6 → SOH6p. The rules for such organism specific expansions must be deduced from a given training set (as it was the case here) or by manual analysis of a set of texts if no annotated training set is available.

In a second step, synonyms matching common English words are removed (this step is not done for fly, which has many valid protein names that are common English words). Synonyms containing subtype specifiers are expanded by the synonym without subtype specifier if there is only one subtype mentioned in the synonym list (aminoacylase 1 → aminoacylase).

The third step accomplishes further expansion and pruning. The tool used for this purpose was provided by D. Hanisch, for a detailed description see [6]. In the expansion phase, new synonyms are added to the existing ones. The expansion is based on rules and lists. A list of frequent acronyms and long names is used for expanding every occurrence of a common acronym in the synonym list to the corresponding long name and reducing long names to acronyms (IL⇔interleukin).

Inappropriate synonyms are detected and removed in the pruning phase by using token-class based regular expressions. A token can be any sequence of letters and/or numbers. A token class is a group of words which have a similar meaning or usage. Examples of token classes are: measuring units (contains: kDa, Da, mg,...), common words (if, and, as, for, ...), descriptions (tRNA, Ser, Tyr,...), numbers, single letters. These token classes are combined in regular expressions, e.g. 'a number followed by a measuring unit', 'one description', 'a common word followed by a number'. Synonyms that are matched exactly by one of these regular expressions are removed, e.g. '22 kDa' is removed by the regular expression 'a number followed by a measuring unit'; 'If 1' is removed by the pattern 'a common word followed by a number'. The lists of words belonging to a token class and the rules for combining them in regular expressions were compiled during previous work (based on analysis of synonyms provided in Swiss-Prot [16] and HUGO [17] and their matching statistics against MEDLINE abstracts). The lists and rules used during the third curation step are of a general character and hence are usually not adapted when applied to new synonym lists. Ambiguous synonyms (i.e. synonyms belonging to more than one protein) generally need to be assigned or disambiguated to one of the corresponding proteins. Our approach does no disambiguation, therefore ambiguous synonyms are removed from the synonym list. Objects which have no synonym left are removed from the synonym list.

The standard curation procedure was adapted to BioCreAtIvE as follows: For yeast, the expansion rule for the extension 'p' was added. For fly, common words were not removed. For all organisms, synonyms that produced many false positive but not true positive matches in the training data were removed. The results on the provided hand-curated training set were analysed manually and some obvious, but missing synonyms were added (about 15 synonyms).

Due to the added spelling variants and expansion of abbreviations the curated synonym lists are significantly larger than the original ones. The curated mouse synonym list contains on average 7.6 synonyms per object compared to 2.5 synonyms per object in the original list. The curated yeast synonym list contains on average 5.2 synonyms per object compared to 1.9 synonyms per object in the original list. The run-time of the entire curation procedure depends on the size of the synonym list and the rules that are applied; it is about 2 minutes for the yeast synonym list.

### Match detection

Synonyms as defined in the synonym list are searched within the texts by exact text matching. The search is case insensitive only if the synonym contains numbers or if the synonym length is above a certain threshold (5 characters). When several synonyms of different length can be matched at a certain text position, only the longest match is reported.

### Rule-based post filter

We implemented a simple rule-based post filter that checks occurrences of synonyms for nearby occurrence of modifiers (e.g. 'cells', 'domains', 'cell type', 'DNA binding site') indicating that the passage of text does not refer to a protein.

Short synonyms in parentheses often overlap with definitions of abbreviations differing from the assumed protein, e.g. '...mapped by fluorescence in situ hybridization (FISH)...', '...developing mouse submandibular gland (SMG)...', 'Fish' and 'SMG' are valid mouse protein names, but the text does not refer to these as proteins. We clarify the meaning of such occurrences by checking the words ahead of parentheses corresponding to the letters of the synonym. If no significant overlap of these words with the alternative names of the assumed protein is found the match is discarded. As example the alternative name for 'Fish' is 'five SH3 domains', for 'SMG' it is 'small nuclear ribonucleoprotein polypeptide G', both have no overlap with the text fragments before parentheses shown above and are therefore removed.

### SVM-based post filter

Fly synonyms show a significant overlap with common English words, body parts and phenotypic descriptions and therefore require context dependent evaluation. After the BioCreAtIvE assessment, a post filter based on support vector machines (SVM) [18] has been implemented.

First, the curated fly synonym list is searched against MEDLINE-abstracts. Matches of multi-word synonyms are always accepted. Matches of single-word synonyms are evaluated by the SVM and classified as true or false hits. The SVM uses the following features:

• surface keys, i.e. orthographic properties of the matched synonym: synonym length; whether it contains non-characters, numbers, greek numbers, capitals, lower-case letters, numbers and letters; whether it consists entirely of capitals, lower-case letters; whether it has a capital after a non-capital; whether the first letter is upper case followed by only lower case

• part of speech tags [19] of the matched synonym and directly adjacent words

• prefix and suffix of the synonym (the first and last 2 and 3 letters)

• all substrings of length 3 of the synonym

The feature value for the synonym length corresponds to the number of characters of the synonym. All other features are encoded as binary values (e.g. one feature is defined for each possible substring of length 3 and the corresponding feature value is set 1 for all substrings that appear in the considered synonym, all other substring feature values are set 0).

Furthermore, we use scores that indicate how often a word was found close to a correct synonym match. Six categories of words were used: nearest verbs, nearest nouns, and words adjacent to a synonym match; occurrences before and after a match were considered separately.

Scores for nouns and verbs were generated from the 5,000 abstracts of the fly training set. We analysed each sentence that contains a synonym and extracted the closest verb and noun [19] before and after the synonym match. As positive samples, we used the correct matches, as negative samples the false matches (false positives). For these verbs and nouns a score is calculated as described below.

A second set of scores is based on a search of mouse synonyms against approximately 700,000 MEDLINE abstracts. In this data set, words appearing adjacent to synonym matches are extracted irrespective of their grammatical class. Since we have no standard of truth for this data set, we assume every match as positive sample and extract the words adjacent to it. In order to estimate the background frequency of words, we consider all words of every sentence of that data set where no synonym has been matched as negative.



These scores are then used as SVM feature values: The directly adjacent words and the closest verbs and nouns before and after a synonym match are extracted. For each category, the score of the word is used as value for the corresponding feature, it is 0 if no score was defined for the word.

The SVM uses a linear kernel. It is trained on 10,000 exact matches of single-word synonyms against the fly training set, where each true hit is used as positive sample and each false hit as negative sample. For the prediction, the curated synonym list is matched against the abstracts of the test set. Matches of multi-word synonyms are accepted directly. Every match of a single-word synonym is classified by the SVM as positive or negative. A single word synonym is only accepted for an abstract if at least one match of this synonym within the abstract is classified as positive. All matches of multi-word synonyms and the accepted single-word synonyms are reported as final result.

In this paper we only show the usability of the SVM-based post filter for fly synonyms. The motivation for using scores obtained by searching fly and mouse synonyms against two different sets of abstracts was to exploit more information than given in the annotated training data.

### Principles of the ProMiner approach

The ProMiner team used essentially the same curated mouse synonym list as we did. Thus BioCreAtIvE allows a comparison of our exact matching approach to the ProMiner approach, which is described in detail in [6,12]. Here we only give a short description of the ProMiner principles.

The match algorithm implemented in ProMiner is based on token classes. Different token classes have different weights according to their relevance for the protein name, e.g. tokens of the class 'Modifier' (class contains tokens like: inhibitor, ligand, antagonist,...) are important and thus have a high weight whereas tokens of the token class 'Description' (contains: chain, component, product,...) have a low weight. This weighting scheme allows that a multi-word synonym is recognized even if certain less relevant parts of it are missing in the text. The ProMiner framework additionally applies different post filters and thus filters out non-specific synonyms. It has disambiguation capability, i.e. depending on the context of a synonym match it can narrow the list of proteins that an ambiguous synonym refers to. The tolerance of matching, the stringency of post filters, and also the accepted level of ambiguity for reporting is defined by a set of parameters that can be tuned for a specific application.

### Evaluation

The Evaluation was performed on 250 MEDLINE abstracts for each of the organisms yeast, mouse, and fly. The results were evaluated by the BioCreAtIvE organizers in terms of:



## Results and Discussion

### General performance

**Figure 1:**
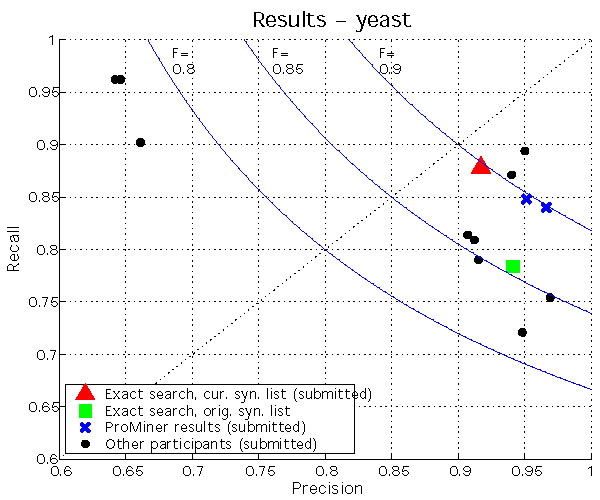
**Yeast results. **BioCreAtIvE task 1B results for yeast and the impact of curation of the synonym list. The submitted result was obtained with the fully curated synonym list.

**Figure 2:**
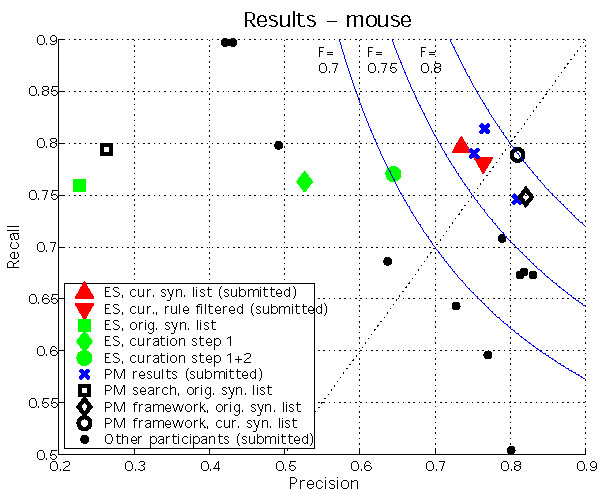
**Mouse results. **BioCreAtIvE task 1B results for mouse and the impact of curation on exact search and the ProMiner approach. ES: Exact search; PM: ProMiner; cur.: fully curated synonym list; orig. syn. list: original synonym list as provided by organizers. For the exact search, the submitted results were obtained with the fully curated synonym list and the fully curated synonym list with subsequent application of the rule-based post filter. The results of exact matching of the original synonym list, and lists obtained from the two intermediate curation steps are also shown. For ProMiner, the results of the approximate search alone (PM search) and the results of the ProMiner framework (i.e. approximate search plus filtering and disambiguation) with optimal parameter setting are shown. The submitted results (PM results) were obtained with the entire ProMiner framework, the same fully curated synonym list and different sets of parameters [12]. The fully curated synonym lists used for exact search and the ProMiner approach were the same except ambiguous synonyms.

**Figure 3:**
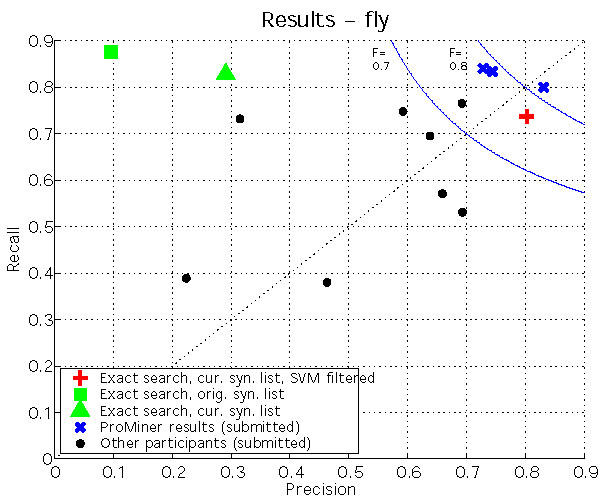
**Fly results. **Results for fly, obtained as post-evaluation of the BioCreAtIvE-assessment. The figure shows the results of exact matching of the synonym list as provided by the ProMiner-team (Exact search, orig. syn. list), exact matching of the curated synonym list and exact matching of the curated synonym list with subsequent application of the SVM-based post filter. All submitted ProMiner results were obtained with one synonym list, which we here refer to as 'original synonym list', but different parameter settings [12].

For yeast, we submitted one result set without post-filtering. For mouse, we submitted two runs: one without any post-filtering (run 1) and one with the rule-based post filter described above (run 2). For both organisms our tool achieved results close to the best overall results. The results are shown in table 1 and figures 1 and 2. The results for fly (obtained as post-evaluation) are shown in figure 3.

For yeast the difference in F-measure from the best result is 2.4%. This difference is mainly due to lower precision (3.3%), but also recall is somewhat lower (1.6%). For mouse, the best result is a run done with ProMiner; the difference with our results in F-measure is 2.6%/1.7%. This run was done with the same synonym list, the only difference being that the list for ProMiner contained ambiguous synonyms, which were removed from our list.

Some examples of errors in the recognition of mouse gene names are listed in the tables 2, 3, and 4. The errors in the yeast results are similar, and are not discussed in detail.

BioCreAtIvE shows the different levels of difficulty for protein name recognition for different organisms; yeast has a quite precise nomenclature consisting mainly of distinctive single word synonyms, compared to mouse with many multi word protein names, and fly for which a large number of synonyms exist that overlap with standard English words and anatomic descriptions.

Our results show that a straightforward approach for protein name recognition can be successful. Exact matching of curated synonyms results in good recall and precision for yeast and mouse, the results are only marginally below those of the best methods available.

Curation and exact matching of fly synonyms results in low precision (figure 3). This pinpoints a limit of the 'simple' approach. The results after application of the SVM-based post filter show that this limit can be overcome by additional application of more involved techniques.

### Curation of synonym list

Figures 1, 2, and 3 show the impact of curation. The result obtained with the original, non-curated, and the final, fully curated, synonym list is shown for all three organisms. The results of the fully curated lists of yeast and mouse were those that were submitted to BioCreAtIvE.

The curation of the yeast synonym list increases recall significantly while precision decreases slightly (Figure 1).

For mouse, figure 2 shows the results for the original and the fully curated list and also results for intermediate curation steps. The figure shows that already an exact search with a list returned from steps 1+2 of our curation procedure yields results which are comparable to those submitted by other groups. The final results were generated by applying all three curation steps. The additional execution of the third step of curation, namely the removal of inappropriate synonyms based on regular expressions of tokens and the expansion of acronyms and long names yields a further increase in recall and precision. The complete curation procedure significantly increases precision and also slightly improves recall of the mouse synonym list.

During the curation procedure, all ambiguous synonyms were removed. We analyzed two scenarios for estimating the effect of not removing them from the mouse synonym list. If we kept all ambiguous synonyms and reported all proteins to which they belong, we would obtain 24 additional correct matches and 133 false matches (recall: 84.0%, precision: 61.2%, F: 70.8%). If we were able to disambiguate them to the correct objects, which would be the ideal case, this would have been 24 additional correct matches and no additional false matches (recall: 84.0%, precision: 74.6%, F: 79.0%).

Figure 3 shows the effect of curation on fly. Precision is significantly increased by curation and recall slightly decreased. The F-measure obtained with the fully curated list is still low (43.1%), which is due to the low precision (29.1%) of matches of synonyms resembling common words and descriptions, a problem that is addressed and largely eliminated by the SVM-based post filter.

### False positives

All false positive matches are correct matches of a valid synonym, they appear as false positives because the occurrence does not refer to the protein that was assumed to be mentioned. In these cases, the context reveals the intended meaning of the expression.

The false positive matches can be classified in different categories. Some examples are listed in tables 2 and 3. Several false positives originate from phenotypic descriptions, e.g. 'growth retarded'. Detailed grammar or semantic analysis would be required to distinguish between such descriptions and the gene being associated with the phenotype. Other false positive matches have keywords close-by that clearly indicate that the match should not be reported because it refers to a different organism or it is not the focus of interest, e.g. 'human *doublecortin*' or '*BMP2*-mediated'. These matches could easily be filtered out by the rule-based post filter, which does not yet consider organisms and words indicating that a match is only a passing mention. The post filter removes several false positive matches and so slightly increases precision, some examples are given in table 3.

### False negatives

The false negative matches can be classified into three groups (see table 4 for some examples): missing synonyms, different spellings of synonyms, and ambiguous synonyms.

In the future, recall could be increased by covering more spelling variants. Some of the false negatives can be recovered by quite simple means such as equal treatment of space and hyphen or a further extension of subtype descriptors (e.g. alpha, a, I, 1). Inversions are more difficult to deal with as they are not always allowed. The inclusion of ambiguous synonyms could also bring about an improvement.

In some cases proteins are mentioned by expressions which have no clear relation to any of the given synonyms. These cases are difficult to handle.

The analysis of the false negative matches of yeast showed that long names of some proteins were used in abstracts while our synonym list contained only the corresponding short names. Some of these long names could have been extracted from description fields of the Saccharomyces Genome Database or Swiss-Prot. We only used the original synonym lists and applied the curation procedure as described above for obtaining the final synonym list. We did not include further information as contained in the database description fields or the list of additional yeast gene descriptions provided by the organizers. The reason for this is that we wanted to evaluate our approach in a way so that it could be applied for a large set of organisms, which possibly are not as well annotated with additional description fields as yeast, mouse and fly. It is certain that by considering further data sources as the annotations and descriptions in organism specific databases or general databases like Swiss-Prot, it will be possible to discover further synonyms and thus obtain higher recall.

### Rule-based post filter

The rule-based post filter was applied on mouse results; it increases precision by 2.9% and decreases recall by 1.5%. This shows that the approach is in principle useful but also shows its limits. The rules applied for filtering out false positives were defined after a crude manual analysis of the results on the training set. Further enhancement is clearly possible.

One of the aims of the BioCreAtIvE evaluation is the organism-specific recognition of gene/protein names. Our approach does not yet include an organism filter. Precision might be increased by disapproving matches that co-occur with organism names distinct from the organism of interest.

The examples of false positive matches in table 2 suggest further rules: All matches with a close-by occurrence of words indicating a passing mention (like '...-mediated','...-activated',...) could be removed; Part of speech tagging could help to identify descriptions like '*striated *muscle', and one could consider removing matches that are tagged as adjective.

A more detailed analysis of false positive matches would probably produce further rules, but this needs intensive manual effort. Another possibility could be the generation of rules by automatic means, e.g. statistic analysis of word frequencies. We propose the usage of the SVM-based post filter instead of the rule-based post filter, as it also considers close-by words but does not need manually generated rules.

### SVM-based post filter

The identification of fly synonyms highlights the limits of the simple approach consisting of extensive curation and exact matching of a synonym list. The nomenclature of fly makes it indispensible to filter matches depending on the context. We used a SVM to filter hits resulting from exact matching.

As descriptors, we use a number of commonly used features, such as surface keys, part-of-speech tags, and substrings. Furthermore, we exploit the capability of our system to recognise mouse synonyms with satisfying accuracy and speed. We estimate scores for words appearing close to synonym matches within a large set of MEDLINE abstracts. These scores indicate the frequency of occurrence of the word with synonym matches. Some examples of the top-ranked words are: interactor, protooncogene, costimulates (category 'word directly after synonym match'); heterodimer, transcripts, corepressor (category 'noun after match'); exerts, suppresses, encodes (category 'verb after match'). These words are strong indicators of a gene-/protein-mention.

Thus, we include more information than given in the original training set. The SVM-based post filter proves to be very effective in filtering matches of fly synonyms, it increases precision by 51.1% and F-measure by 33.7% compared to the exact matching of the curated synonym list without post-filtering. The analysis of the filtered matches of the evaluation data set showed that most synonyms were either never (e.g. modulo, rough, snake, forked) or always (e.g. to, for, key, gel, lines) filtered, and that this is almost always correct according to the annotation of the organizers. In some cases context is crucial for correctly classifying results, e.g. the word 'torpedo' in '... the signals transduced by the torpedo product ...' describes a fly gene, whereas in '... the mature Drosophila AChE is closely homologous to that of Torpedo AChE.' it describes an organism. These mentions were correctly classified by the SVM-based post filter. The filter also has a positive effect on the matches of yeast and mouse synonyms (results not shown). A significant advantage of this filtering approach compared to the rule-based post filter is its independence of manually generated rules and its general applicability.

### Comparison to approximate matching implemented in ProMiner

Our results show that especially for organisms having a stringent terminology, such as yeast, exact text matching is useful and reasonable for protein name recognition. For such organisms, an approximate search like the algorithm applied in ProMiner does not improve the results significantly. The results for mouse show that for organisms with a more difficult terminology there is a slight difference in performance between exact text matching and approximate search. Considering the best submitted results of both approaches (those yielding highest F-measure), precision is similar but recall is higher for approximate search. Keeping in mind the approximate matching procedure of ProMiner, this is obvious.

The result of the basic ProMiner search with the non-curated synonym list and no filtering and disambiguation (Figure 2, PM search, orig. syn. list) is slightly better than the results of exact matching of the non-curated synonym list. This is due to approximate matching and the internal scoring function that eliminates poor matches. The full ProMiner framework includes extensive filtering and disambiguation. With optimal parameter setting this system shows good results even when using the non-curated synonym list (F-measure 0.78, PM framework, orig. syn. list). The parameters used for this run were acquired during post evaluation and turned out to yield better results than the parameters used for the BioCreAtIvE submissions. By using the curated synonym list with the same settings (PM framework, cur. syn. list) the F-measure increases further to 0.80. This shows that also for an approximate and advanced approach like ProMiner the curation of the synonym list has a significant effect on the search result. There are important advantages of the exact matching procedure: It is easy to run as it does not need any parameter optimisation. As the curation of the synonym list is independent of the search, an iterative curation procedure can be established. This is useful if the search result on a training set indicates bad synonyms which should be removed from the synonym list. The runtime of the curation procedure depends largely on the size and characteristics of the synonym list. For yeast, the curation takes about 2 minutes and the exact search against the training set of 5,000 abstracts including analysis and report of results takes about 45 seconds on a standard machine. The exact search script is implemented in Perl, it has less than 750 lines of code and is easy to adapt to different input and output formats.

ProMiner is less dependent on the curation of the synonym list and is capable of synonym disambiguation, but it is more difficult to set up and handle. The system needs adjustment of different matching parameters which have a significant effect on the results. It needs about 1.5 minutes for preprocessing (i.e. tokenization of synonyms, analysis for token classes and organisation in a search structure) of the yeast synonym list. The search on the corresponding training set including filtering and report of results takes 3.5 minutes.

## Conclusion

Our goal in participating in the BioCreAtIvE evaluation was to assess the recall and precision that can be achieved with extensively curated synonym lists and exact string-matching, and to assess the difference with more sophisticated text-mining approaches. Our mouse synonym list was also used by group 16 with a more sophisticated search algorithm implemented in the tool ProMiner [6,12]. This evaluation allowed us to compare the different approaches on a blind prediction basis and for an independent test set. The results show that the difference in terms of recall and precision is small. Our approach showed good performance in protein name recognition with exact text matching. Our system does not need to be adapted for specific synonym lists in terms of parameter tuning or internal lists. This allows for straightforward application. It is crucial for our approach to use synonym lists which are as complete and correct as possible. Therefore, we used a system for the extensive curation of protein synonym lists, based on the application of individual, fully automated steps. This curation is largely independent of the synonym list to be curated since the individual curation steps are of general character. Nevertheless, the system can easily be adapted to cover specific problems of synonym lists, like missing synonyms that are frequently used in texts and which can be deduced from the synonyms in the list by application of rules.

One disadvantage of the extensive curation is the fact that the synonym lists become very large as they need to cover all possible different spellings of a protein name. In order to avoid this, one could consider making the text search more flexible, e.g. by including certain equivalent expressions like space and hyphen directly in the search tool.

Our approach relies heavily on synonym lists and is therefore presumably less useful for applications where such a list is not readily available, e.g. the recognition of general gene and protein names without normalization as in BioCreAtIvE task 1A (in which we did not participate). One could consider generating a general synonym list by joining annotations from a large number of public databases for applying our approach to this task.

BioCreAtIvE clearly demonstrated the different levels of difficulty for identifying gene names of different organisms. For yeast, we obtained good results without any post-filtering, mouse results were slightly inferior, and fly results without post-filtering are unsatisfactory because of low precision. The usage of post filters can compensate for low precision, especially the proposed SVM-based post filter proved to be very effective.

Exact matching of the curated synonym list returns hits with high recall. Depending on the characteristics of the synonym list, an appropriate post filter can be set up individually for increasing precision. The fly results show that also for organisms with an unspecific nomenclature, our approach including post-filtering yields good results.

The separation between matching and filtering allows flexibility in the kind of filter applied, and also makes it possible to gear the final result towards recall or precision.

## Figures and Tables

**Table 1:** Results in BioCreAtIvE Task 1B: Our results compared to results with highest overall F-measure. For mouse and fly the highest F-measure is achieved by ProMiner. Our yeast result was obtained by exact matching of the curated list, no post filter was applied. Mouse(1) is the exact search with the curated list. Mouse(2) was additionally filtered with the rule-based post filter. Our fly results were obtained as post-evaluation by exact matching of the curated list and application of the SVM-based post filter.

	Yeast	Yeast max.	Mouse (1)	Mouse (2)	Mouse max.	Fly (post-eval.)	Fly max.
F-measure	0.897	0.921	0.764	0.773	0.790	0.768	0.815
Precision	0.917	0.950	0.735	0.764	0.766	0.802	0.831
Recall	0.878	0.894	0.796	0.781	0.814	0.737	0.800

TP	538	548	433	425	443	316	343
FP	49	29	156	131	135	78	70
FN	75	65	111	119	101	113	86

**Table 2:** False positive matches: Types of errors and samples. Synonyms are marked in *italics*. The synonyms and matches are correct but the context reveals that they should not have been reported for BioCreAtIvE task 1B.

Type of error	Examples
overlap with English Words wrong organism no direct mention of protein description of different object synonym has different meanings	*striated *muscle, *killer *cells, *Low *effectiveness... Mutations in the human *doublecortin* ... inhibits *BMP2*-mediated induction of ... ... with the *androgen receptor *antagonist cyproterone acetate ... ... transgenic mice are *growth retarded*, ... is required for normal *cardiac morphogenesis*

**Table 3:** Rule-based post filter: Samples of false positive matches, mostly short names and abbreviations of protein names which have different meanings, and the effect of the rule-based post filter on these matches.

Synonym	Context	Other synonym for wrongly identified object	Removed by post filter
P21	Chromosome 2p16-p21	cyclin-dependnet kinase inhibitor 1A (P21)	no
FACS	fluorescence-activated cell sorter (FACS)	fatty acid Coenzyme A ligase, long chain 2	yes
PCR1	E. coli plasmid pCR1	mannosidase 1, alpha	no
CA1	area CA1 of the hippocampus	carbonic anhydrase 1	no
HEK	HEK cells	Eph receptor A3	yes
NT2	NTera 2(NT2) cell line	zinc finger protein 263	yes
Eph	Eph family of receptors	Epa receptor A1	no
PMN	polymorphonuclear (PMN) infiltration	progressive motor neuropathy	yes
all-trans	All-trans retinoic acid	retinol dehydrogenase 2	no
slp	sphingosine 1-phosphate receptor genes	site-1 protease	no
Den	diethylnitrosamine (DEN)	denuded	yes

**Table 4:** Samples of false negative matches: closest synonyms in synonym list, occurrence in text, and type of error.

Synonym(s)	Occurrence in text	Type of error
Lpa1, Lpa2, Lpa3	lpa(1-3)	enumeration
Pkcb, Pkce	PKC beta, PKC-epsilon	different spelling
retinoic acid receptor, alpha	retinoic acid receptor-alpha	different spelling
interferon gamma	gamma-interferon	inversion
Braf2, Braf-rs1	Braf	ambiguity
peroxisome proliferator activated receptor gamma	peroxisome proliferating antigen receptor gamma	not evident
